# Hsa-miR-323a-3p functions as a tumor suppressor and targets STAT3 in neuroblastoma cells

**DOI:** 10.3389/fped.2023.1098999

**Published:** 2023-03-24

**Authors:** Swapnil Parashram Bhavsar, Lotte Olsen, Cecilie Løkke, Jan Koster, Trond Flægstad, Christer Einvik

**Affiliations:** ^1^ Pediatric Research Group, Department of Clinical Medicine, Faculty of Health Science, UiT—The Arctic University of Norway, Tromsø, Norway; ^2^ Department of Oncogenomics, Center for Experimental and Molecular Medicine (CEMM), Amsterdam University Medical Centers, University of Amsterdam, Amsterdam, Netherlands; ^3^ Division of Child and Adolescent Health, Department of Pediatrics, UNN–University Hospital of North-Norway, Tromsø, Norway

**Keywords:** non-coding, microRNAs, neuroblastoma, chromosome region 14q32, chemotherapy, STAT3

## Abstract

**Background:**

Studies conducted in the last decades have revealed a role for the non-coding microRNAs (miRNAs) in cancer development and progression. Several miRNAs within the chromosome region 14q32, a region commonly deleted in cancers, are associated with poor clinical outcome in the childhood cancer neuroblastoma. We have previously identified *miR-323a-3p* from this region to be downregulated in chemotherapy treated neuroblastoma cells compared to pre-treatment cells from the same patients. Furthermore, in neuroblastoma tumors, this miRNA is downregulated in advanced stage 4 disease compared to stage 1–2. In this study, we attempt to delineate the unknown functional roles of *miR-323a-3p* in neuroblastoma.

**Methods:**

Synthetic miRNA mimics were used to overexpress *miR-323a-3p* in neuroblastoma cell lines. To investigate the functional roles of *miR-323a-3p,* cell viability assay, flow cytometry, reverse transcription-quantitative polymerase chain reaction, luciferase reporter assay and western blot were conducted on the neuroblastoma cell lines Kelly, SH-SY5Y and SK-N-BE(2)-C.

**Results:**

Ectopic expression of *miR-323a-3p* resulted in marked reduction of cell viability in Kelly, SH-SY5Y and SK-N-BE(2)-C by causing G1-cell cycle arrest in Kelly and SH-SY5Y and apoptosis in all the cell lines tested. Furthermore, mRNA and protein levels of signal transducer and activator of transcription 3 (*STAT3*) were reduced upon *miR-323a-3p* overexpression. A direct binding of the *miR-323a-3p* to the 3′UTR of *STAT3* was experimentally validated by luciferase reporter assay, where *miR-323a-3p* reduced luminescent signal from full length *STAT3* 3′UTR luciferase reporter, but not from a reporter with mutation in the predicted seed sequence.

**Conclusions:**

*miR-323a-3p* inhibits growth of neuroblastoma cell lines through G1-cell cycle arrest and apoptosis, and the well-known oncogene *STAT3* is a direct target of this miRNA.

## Introduction

Neuroblastoma is one of the most common embryonal malignancies among children and 40% of all children diagnosed with neuroblastoma are designated as high-risk patients (1) with poor clinical outcome (2). Multiple treatment modalities are available, including intensive chemotherapy with autologous stem-cell rescue, surgery, radiation, and immunotherapy, which have improved the survival rate of high-risk neuroblastoma patients. However, many high-risk patients ultimately relapse and eventually die from disease progression. Treatment failure is mainly attributed to the development of drug resistance and is one of the major clinical obstacles in treatment of high-risk neuroblastoma (1, 3). Thus, development of more effective targeted therapies is required to address this issue.

MicroRNAs (miRNAs) are evolutionary conserved, endogenously expressed, small non-coding RNAs (∼19–24 nucleotides) that regulate gene expression by translation inhibition or degradation of mRNA. They are thus responsible for regulating the expression of genes involved in a myriad of cellular processes (4). Recently, miRNAs are shown to modulate drug resistance in multiple cancers (5). Some researchers have identified a differential expression of miRNAs in parental (chemo-sensitive) vs. resistant (chemo-resistant) cancer cells (6, 7). Interestingly, some molecular mechanisms underlying drug resistance have also been elucidated (8).

The *MIR-323a* gene is located on the chromosome region 14q32, a region frequently dysregulated in cancers (9–12). Several miRNAs from this cluster have been found downregulated in neuroblastoma cells from patients with relapsed disease (7). An aberrant expression of *miR-323a-3p* is observed in multiple cancers. *MiR-323a-3p* was downregulated in glioblastoma (13), osteosarcoma (14), pancreatic ductal adenocarcinoma (PDAC) (15), breast cancer (16), colorectal cancer (17) and bladder cancer (18). Whereas in prostate cancer, *miR-323* was upregulated and promoted cell proliferation (14, 19). The role of *miR-323a-3p* in neuroblastoma is unknown and investigation is warranted given the important role of this miRNA in other cancers.

STAT3 is one of seven members (STAT1, STAT2, STAT3, STAT4, STAT5A, STAT5B and STAT6) of the signal transducer and activator of transcription (STAT) protein family (20, 21). This protein was initially shown to be activated in response to binding of cytokines and growth factors to cellular receptors, which activates membrane-associated janus kinases (JAK). JAK in turn phosphorylates STAT3 at specific residues to form homo/heterodimers and translocate to the cell nucleus. In the nucleus, STAT3 acts as a transcription factor, regulating the expression of a wide range of genes involved in survival, proliferation, invasion, metastasis, angiogenesis, and immunosuppression (22). Zhou C. and colleagues have shown that downregulation of STAT3 induces G1-cell cycle arrest and apoptosis in esophageal carcinoma (23). Accumulating evidence suggests that STAT3 is activated by numerous activators (e.g., cytokines, growth factors, toll-like receptors, etc.) (22). Hence, dysregulation of *STAT3* can lead to oncogenesis through various mechanisms. Several studies have identified different miRNAs having reciprocal interactions with JAK-STAT3 signaling pathway in different cancer types, for example, *let-7* (24), *miR-9* (25), *miR-337–3p* (26), *miR-26a* (27) and *miR-135a* (28). However, the role of miRNA directly targeting *STAT3–3*′UTR is not yet demonstrated in neuroblastoma.

We have previously observed downregulation of *miR-323a-3p* in post-chemotherapy neuroblastoma cell lines as compared to matched pre-chemotherapy neuroblastoma cell lines (7). In this study, we set out to understand the functional role of *miR-323a-3p* in neuroblastoma. Therefore, gain-of-function studies were set up by overexpressing *miR-323a-3p* in neuroblastoma cell lines*,* which had significant effect on growth and survival by inducing G1-cell cycle arrest and apoptosis. Furthermore, we demonstrate *STAT3* as a novel target of *miR-323a-3p*.

## Materials and methods

### Cell lines and cell culture

The neuroblastoma cell lines Kelly, SH-SY5Y and SK-N-BE(2)-C [BE(2)-C] were all maintained at 37°C in RPMI-1640 medium with 2 mM L-Glutamine (Sigma-Aldrich) supplemented with 10% fetal bovine serum (Sigma-Aldrich), in a humidified incubator with 5% CO_2_ atmosphere. In collaboration with the Center of Forensic Genetics (UiT—The Arctic University of Norway, Norway), we authenticated the cell lines using short tandem repeat (STR) profiling. We confirmed absence of mycoplasma contamination in the cell lines using MycoAlert^TM^ Mycoplasma Detection Kit (Lonza).

### Transfections

For ectopic expression, 25–40 nM of *miRNA-323a-3p* or negative control (NC) mirVana® miRNA mimics (Ambion, Thermo Fisher Scientific) were transfected using Invitrogen™ Lipofectamine™ 2000 Transfection Reagent (Fisher Scientific) in OptiMEM medium (Thermo Fisher Scientific) according to the manufacturer's instructions.

### Cell viability

Cell viability was assessed using alamarBlue® (Thermo Fisher Scientific) cell viability assay according to the manufacturer's instructions. 25 nM *miR-323a-3p* or NC mimics were reverse transfected into Kelly, SH-SY5Y and BE(2)-C cells seeded in 24-well plates. Cell viability at 24, 48, 72 and 96 h after transfection were measured in a CLARIOstar microplate reader (BMG LABTECH) and calculated as the percentage of NC transfected cells set to 100%.

### Flow cytometry

For determining the cell cycle distribution, Kelly, SH-SY5Y and BE(2)-C cells were first reverse transfected with 25 nM *miR-323a-3p* or NC mimics in 25 cm^2^ culture flasks. After 72 h, the cells were detached by trypsin, centrifuged, and washed with 1 × phosphate-buffered saline (PBS). An overnight incubation at −20°C in 70% ethanol was performed to fixate the cells. After 10 min centrifugation at 850 g, and a subsequent wash with 1 × PBS, fixated cells were added DNA-staining solution consisting of PBS with 50 µg/ml propidium iodide (PI) and 100 µg/ml RNase (Life technologies). Cells were protected from light and stored on ice during a 30 min incubation period prior to flow cytometry measurement of PI-stained DNA in a BD LSRFortessa^TM^ cell analyzer (BD Bioscience). The Dean-Jett-Fox model for cell cycle evaluation was used for analysis in the FlowJo 7.6.5 software.

### MicroRNA target prediction

A computational approach with miRDB algorithm (available at http://www.mirdb.org/) was used to identify *miR-323a-3p* targets (29).

## RNA Extraction, Reverse Transcription and Quantitative PCR

Kelly, SH-SY5Y and BE(2)-C cells were transfected with 25 nM *miR-323a-3p* or NC mimics in 6-well plates. The QIAzol® Lysis Reagent (QIAGEN) was used for isolation of total RNA 24 h later according to the manufacturer's instructions. Quantity and purity of total RNA was assessed with NanoDropTM 2000 spectrophotometer (Thermo Fisher Scientific).

For miRNA and mRNA expression analysis, complementary DNA (cDNA) synthesis from total RNA, and successive quantitative polymerase chain reaction (qPCR)-measurements, were performed as previously described (30). The qPCR cycling was carried out in a Light Cycler 96 SW 1.1 (Roche). For miRNA analysis the miScript primer assays Hs_miR-323–3p_2 (cat. no MS00037219) and Hs_miR-4286_1 (cat. no MS00021371) (QIAGEN) were used for *miR-323a-3p* quantification and as a reference gene, respectively. Using the web-based versions of the LinRegPCR program (https://www.gear-genomics.com/rdml-tools/) (31), we generated mean PCR efficiency for each amplicon group. Expression of *miR-323a-3p* relative to *miR-4286* was calculated as: Expression = E(GOI)^ -Cq(GOI)/E(REF)^ -Cq(REF) (E, PCR efficiency; GOI, gene of interest (*miR-323a-3p*); REF, reference gene (*miR-4286*)) (32). The following primers were used for mRNA expression analysis: *STAT3* (forward: 5′-CAG CAG CTT GAC ACA CGG TA-3′; reverse: 5′- AAA CAC CAA AGT GGC ATG TGA -3′), *BCL2* (forward: 5′-TCG CCC TGT GGA TGA CTG A-3′; reverse: 5′- CAG AGA CAG CCA GGA GAA ATC AA-3′) and *SDHA* (forward: 5′-CTG ATG AGA CAA GAT GTG GTG-3′; reverse: 5′-CAA TCT CCC TTC AAT GTA CTC C-3′). *SDHA* functioned as a reference gene. Expression of *STAT3* and *BCL2* was assessed by the *ΔΔ*Cq comparative cycle threshold method according to Taylor et al. 2019 (33).

All reverse transcription (RT)-qPCR reactions were performed in triplicates on at least three independent biological replicates. For the mRNA expression analysis, the Student's unpaired t-test was used to calculate statistical differences between *Δ*Cq values of NC-treated and *miR-323a-3p*-treated cell lines. Values are presented as the mean normalized expression ± standard error (SEM).

### Western blot analysis

Cells were seeded in 6-well plates, and, 72 h later, trypsinized and lysed in 40 µl RIPA buffer (50 mM Tris-HCL pH 8, 150 mM NaCl, 1% NP-40, 0.5% sodium deoxycholate, 0.1% SDS) containing 1 × Protein Inhibitor Cocktail (Roche) and 1 mM dithiothreitol (DTT) (Sigma-Aldrich). For PARP-cleavage analysis, floating cells were included. Total protein concentrations were determined using DC^TM^Protein Assay Kit (Bio-Rad) according to the manufacturer's instructions, and 40 µg protein was separated on a NuPAGE® Novex 4%–12% Bis-Tris precast polyacrylamide gel (Thermo Fisher Scientific) before blotted onto Immobilon-FL PVDF membrane (Millipore). Prior to fluorescence detection, the membrane was blocked for 1 h at room temperature in 5 ml Odyssey Blocking Buffer (LI-COR Biosciences) followed by overnight incubation at 4°C with primary antibodies: Stat3 (C-20): sc-482, rabbit, polyclonal (1:1000) (Santa Cruz Biotechnology); PARP: 9542, rabbit, polyclonal (1:1000) (Cell Signaling Technology); BCL2 (C-2): sc-7382, mouse, monoclonal (1:200) (Santa Cruz Biotechnology) and Anti-Actin antibody [ACTN05 (C4)]: ab3280, mouse, monoclonal (1:1000) (Abcam). After four (5 min) washes with 0,1% PBST, the membrane was incubated with secondary antibodies Rabbit IgG (H&L) Antibody DyLight™ 800 Conjugated (1:5000) (Rockland Immunochemicals) and goat anti-mouse-Alexa Fluor 680 (1:5000) (Thermo Fisher Scientific) and scanned in the Odyssey CLx Infrared Imaging System (LI-COR Biosciences). Actin was used as loading control. For quantification of protein, the ImageJ software was used (34) (available on imagej.net).

### Reporter constructs and dual luciferase assay

The cells were grown on a 12-well plate and co-transfected with 40 nM mimics, 50 ng/ml pMIR-Report-Firefly construct (Ambion) and 100 ng/ml mutated (pLightSwitch-STAT3–3′UTR-mut) or wild-type (pLightSwitch-STAT3–3′UTR-wt) luciferase constructs harboring full-length *STAT3–*3′UTR.

The pLightSwitch-STAT3–3′UTR-wt construct was obtained from SwitchGear Genomics (Product ID: S813664). pLightSwitch-STAT3–3′UTR-mut construct with a mutation in the putative *miR-323a-3p* seed sequence was generated using QuickChange II Site-Directed Mutagenesis kit (Agilent Technologies). The primers used for mutagenesis were: Forward: 5′-CTG CCC AGC CTT ACT CAC TAA AAG GCC AAT AGC GGA CAA AGG AAA ATA AGT CTA TTT ATA A -3′; reverse: 5′-TTA TAA ATA GAC TTA TTT TCC TTT GTC CGC TAT TGG CCT TTT AGT GAG TAA GGC TGG GCA G -3′. To confirm mutation in the seed sequence, the mutant plasmid was sequenced using sequencing primer 5′- GAA ACG GGC TTC AGG TCA AAC CC-3′.

After incubation for 24 h at 37°C, luciferase activity was measured using the Dual-Luciferase Reporter Assay (Promega), according to the manufacturer's instructions. The renilla luciferase activity was normalized to the firefly luciferase activity.

### MicroRNA expression data

A subset of 226 primary neuroblastoma tumors, referred to as NRC-226 dataset, from the “Tumor Neuroblastoma NRC Compendium-NRC-364-mirg” miRNA dataset was used to obtain miRNA expression data. Table 1 summarizes the characteristics of the NRC-226 dataset. The dataset was generated using multiplex RT-qPCR assays and consists of tumors from the Neuroblastoma Research Consortium (NRC), a collaboration between several laboratories in Europe. The NRC-226 cohort consists of 55 tumors from Essen, 39 from Ghent, 92 from Amsterdam and 40 from Dublin. The R2: Genomics Analysis and Visualization Platform (http://r2.amc.nl) was used to generate Kaplan-Meier overall survival curves for patients with high and low expression of *miR-323a-3p*.

**Table 1:** Characteristics of primary neuroblastoma tumors from NRC-226 dataset.

Characteristics:	No. of tumors:	%
**INSS tumor stage:**
Stage 1	40	17.7
Stage 2	32	14.2
Stage 3	30	13.3
Stage 4	95	42.0
Stage 4s	29	12.8
***MYCN* amplification:**
Yes	40	17.7
No	186	83.3
**Overall survival:**
Event	60	26.5
No event	166	73.5
***miR-323a* expression data available:**
Yes	195	86.3
No	31	13.7
**Age at diagnosis:**
<18 months	112	49.6
>18 months	114	50.4
**Gender:**
Male	98	43.4
Female	85	37.6
Nd	40	17.7
Total:	226	100

### Statistical analysis

The associations between different characteristics of the cohort, including *miR-323a-3p* expression, and patient overall survival were calculated using a univariable Cox model (Table 2). Characteristics with *p*-values less than 0.05 were considered statistically significant and used in a multivariable cox regression model to evaluate independent predictors of survival. *MiR-323a-3p* was analyzed as a continuous variable using z-scores from R2. The coxph function from the R (v. 4.2.2) package “survival” (v. 3.5) was used to perform the univariable and multivariable Cox regression analyses (35). The proportional hazard assumption was tested using the Schoenfeld residual method implemented by cox.zph function in the survival package. The variable “Age at diagnosis” violated this assumption (i.e., non- proportionality, *p*-value = 0.0067) and the final Cox model was stratified by this variable.

**Table 2:** Cox regression analysis for *miR-323a-3p* in neuroblastoma tumor dataset. Univariable and stratified multivariable Cox regression analysis of the correlation between *miR-323a-3p* expression and clinical features for overall survival in the NRC-226 dataset.

Variable:	Univariable:		Multivariable:	
	HR (95% CI)	*p* value	HR (95% CI)	*p* value
***miR-323a-3p*:** continuous z-scores	0.69 (0.53–0.88)	0.003^**^	1.00 (0.78–1.27)	0.987
***MYCN* amplification (MNA):** 0 = non-MNA,1 = MNA	7.06 (4.19–11.9)	1.98e–13^***^	2.38 (1.30–4.36)	0.0052^**^
**Stage1/2/4s vs. Stage3/4:** 0 = Stage1/2/4s, 1 = Stage3/4	24.80 (6.05–101.8)	7.05e–08^***^	14.02 (3.22–61.05)	0.00043^***^
**Age at diagnosis:** 0= < 18 m, 1= > 18 m	4.94 (2.70–9.03)	2.07e–07^***^	^a^
**Gender:** 0 = Female, 1 = Male	1.10 (0.64–1.90)	0.735	^b^

HR, hazard ratio; CI, confidence interval.

^a^ Stratified in final model.

^b^ Not included due to non-significant in univariable analysis. Significance codes: * = “<0.05′, ** = “<0.01, *** = “<0.001′.

The GraphPad Prism (version 5.00) software for Windows (GraphPad Software) (available at www.graphpad.com) was used for all statistical analyses unless stated otherwise. Analyses are based on at least three independent experiments and presented as mean ± standard deviation (SD). Student's t-test was used to calculate statistical differences between means (*n* = 3) of control and treated cells. *P*-values *P < 0.05, **P < 0.01, ***P < 0.001 were considered to indicate statistically significant results.

## Results

### miR-323a-3p is differentially expressed in neuroblastoma cell line pairs and primary tumors

**Figure 1:**
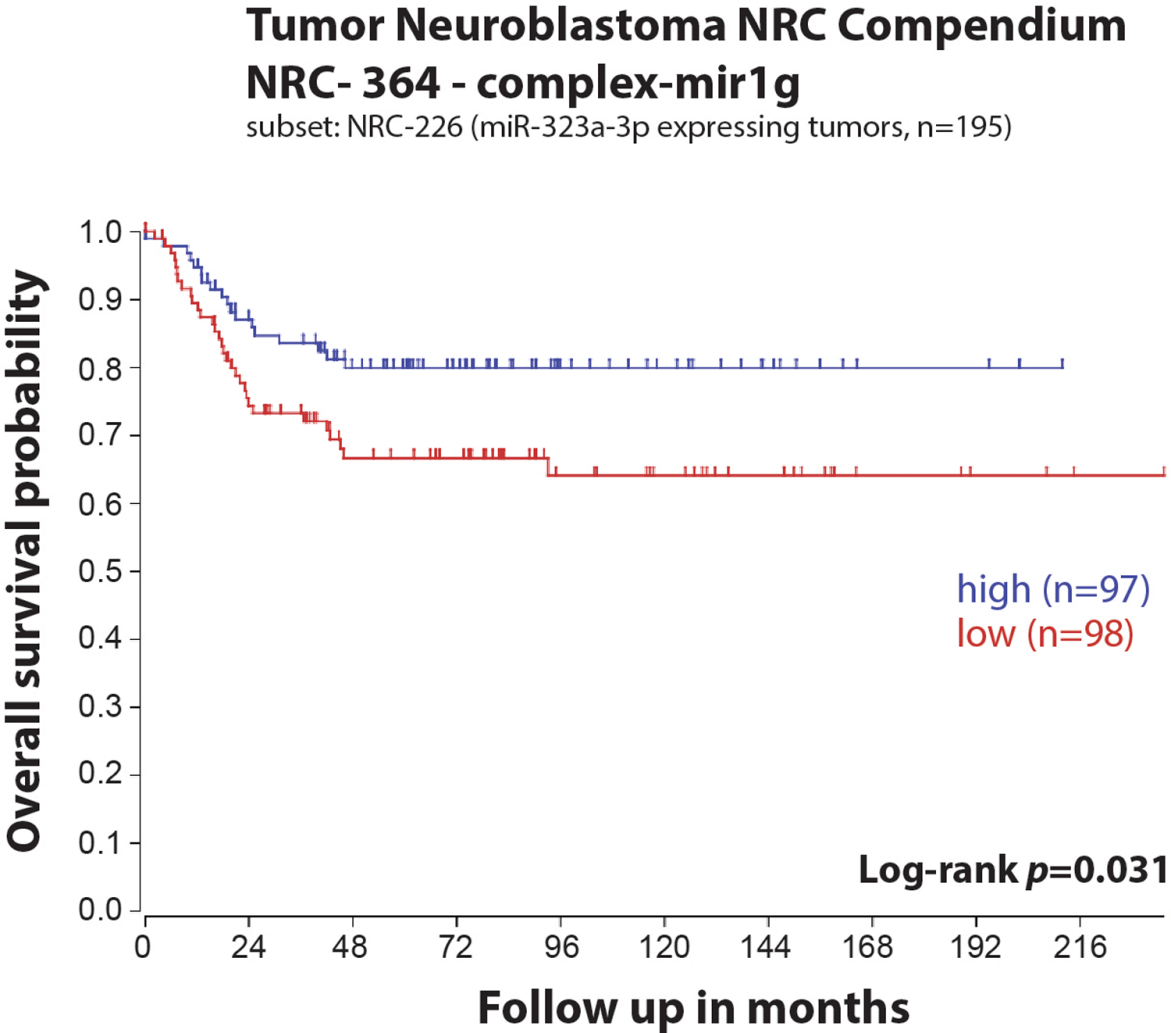
Low expression of *miR-323a-3p* is associated with poor overall survival in a primary neuroblastoma tumors dataset. Kaplan-Meier overall survival curve for patients with high (blue, *n* = 97) and low (red, *n* = 98) expression of *miR-323a-3p*.

In our previous study, we reported a reduced expression of 22 miRNAs from the chromosome 14q32 miRNA cluster (including *miR-323a*) in post-chemotherapy neuroblastoma cell lines as compared to matched pre-chemotherapy neuroblastoma cell lines (Supplementary Figure S1) (7). We showed that *miR-323a-3p* expression was reduced in advanced stage 4 tumors as compared to stage 1–2 and in *MYCN*-amplified (MNA) tumors as compared to non-MNA, two well-known prognostic factors of neuroblastoma (7). When neuroblastoma tumor data from the Neuroblastoma Research Consortium (NRC) was analyzed using Kaplan-Meier method, we observed a significant association between low expression of *miR-323a-3p* and a poor overall survival (Figure 1). Furthermore, we assessed the prognostic effect of *miR-323a-3p* expression and different clinical characteristics using a univariable Cox proportional hazards regression model. The results revealed that *miR-323a-3p*, *MYCN* amplification, age at diagnosis > 18 months, and INSS Stage1,2,4S vs. Stage 4 were associated with overall survival (*p*-value < 0.05) (Table 2). The gender was excluded in the subsequent multivariable analysis since it was not associated with overall survival (*p*-value > 0.05). As expected, the multivariable cox regression analysis showed that the well-established prognostic factors *MYCN* amplification and INSS stage 4 were independently associated with poor survival. However, *miR-323a-3p* expression was not an independent prognostic factor associated with overall survival in the NRC-226 cohort (Table 2).

Others have reported that *miR-323a-3p* is downregulated and acts as a tumor suppressor in various cancers (15, 18). Given these findings, we sought out to elucidate *miR-323a-3p* functional role in aggressive neuroblastoma.

### miR-323a-3p inhibits growth and survival of neuroblastoma cells

**Figure 2:**
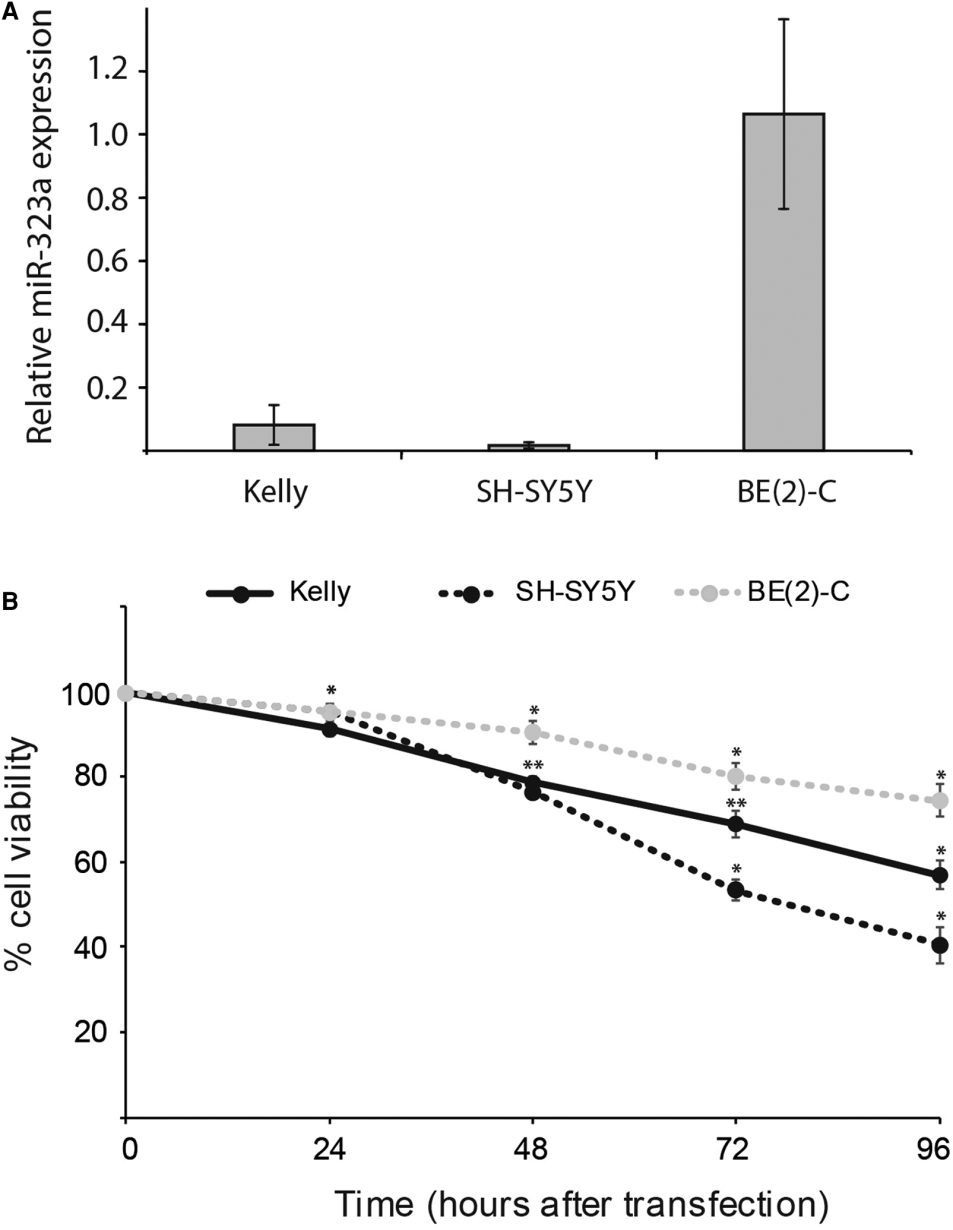
*miR-323a-3p* overexpression suppressed the growth and survival of neuroblastoma cells. **(A)** The basic expression of *miR-323a-3p* with respect to stably expressed *miR-4286* in Kelly, SH-SY5Y and BE(2)-C cell lines were detected by RT-qPCR analysis. Data is presented as mean ± SEM of at least three independent experiments, each repeated in triplicates. **(B)** Cell viability of the cell lines Kelly, SH-SY5Y and BE(2)-C at 24 h, 48 h, 72 h and 96 h post transfection with *miR-323a-3p*, measured with the alamarBlue cell viability assay. Data is presented as mean ± SD of at least three independent experiments, each repeated in triplicates. *P < 0.05, **P < 0.01 vs. the NC. RT-qPCR, reverse transcription-quantitative polymerase chain reaction; NC, negative control; miR, microRNA; SD, standard deviation.

To examine the relationship between reduced expression of *miR-323a-3p* and cell survival, we first evaluated the basic expression of *miR-323a-3p* relative to endogenous *miR-4286* in Kelly, SH-SY5Y and BE(2)-C neuroblastoma cell lines by RT-qPCR. *MiR-4286* is stably expressed in these neuroblastoma cell lines, as reported previously (7). We observed very low levels of *miR-323a-3p* in Kelly and SH-SY5Y as compared to BE(2)-C cell line (Figure 2A).

Next, to check the transfection efficiency, we transfected Kelly, SH-SY5Y and BE(2)-C cell lines with negative control (NC) or *miR-323a-3p* miRNA mimics. RT-qPCR analysis demonstrated that the expression of *miR-323a-3p* was significantly increased in *miR-323a-3p* transfected cells compared to NC transfected cells (Supplementary Figure S2). However, the BE(2)-C cell line had lower transfection efficiency than Kelly and SH-SY5Y.

The overexpression of *miR-323a-3p* in Kelly, SH-SY5Y and BE(2)-C cell lines caused markedly lower cell viability than in NC transfected cells as measured by alamarBlue cell viability assay performed at 24, 48, 72 and 96 h post-transfection (Figure 2B). Collectively, these results demonstrated that overexpression of *miR-323a-3p* affects the growth of neuroblastoma cells.

### miR-323a-3p affects G1-cell cycle arrest and apoptosis in neuroblastoma cells

**Figure 3:**
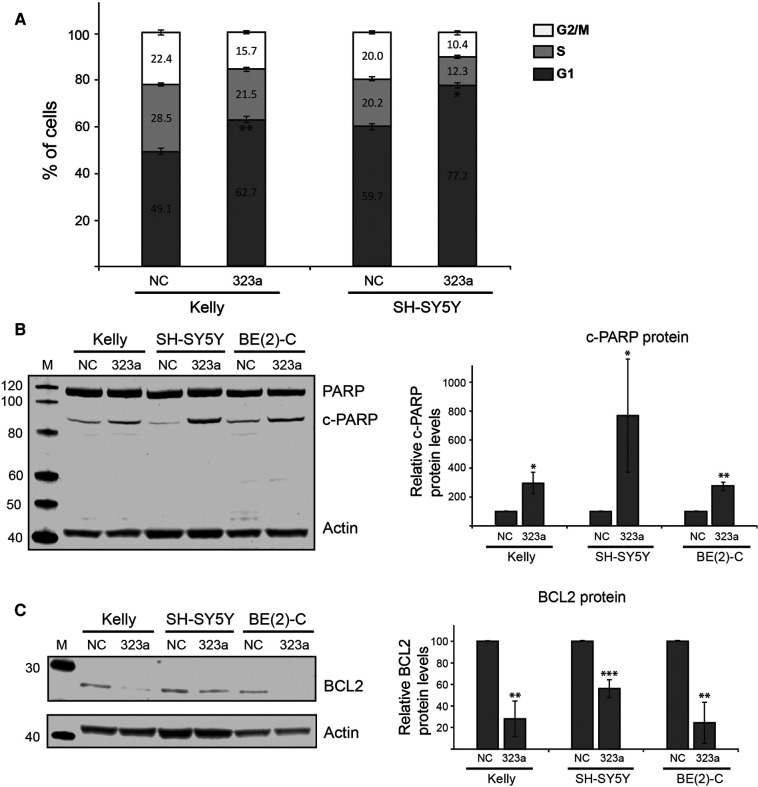
*miR-323a-3p* induces G1-cell cycle arrest and apoptosis in neuroblastoma cells. **(A)** Kelly and SH-SY5Y cell lines were transfected with *miR-323a-3p* or NC mimics and cell cycle distribution was measured by flow cytometry assay. Data is presented as mean ± SD of three independent experiments. **(B)** Total PARP and PARP-cleavage (represents apoptosis) was detected on western blot in Kelly, SH-SY5Y and BE(2)-C cell lines transfected with *miR-323a-3p*. Quantification of cleaved PARP protein levels on the western blots (*n* = 3). (**C**) **(B)** BCL2 was detected on western blot in Kelly, SH-SY5Y and BE(2)-C cell lines transfected with *miR-323a-3p*. Quantification of BCL2 protein levels on the western blots (*n* = 3). Data is presented as mean ± SD of three independent experiments. *P < 0.05, **P < 0.01, ***P < 0.001 vs. the NC. SD, standard deviation; NC, negative control; miR, microRNA; c-PARP, cleaved PARP.

As cell growth is associated with the cell cycle, we analyzed the effect of *miR-323a-3p* overexpression on the cell cycle distribution in Kelly, SH-SY5Y and BE(2)-C by flow cytometry assay. Whereas *miR-323a-3p* expression did not affect cell cycle distribution in BE(2)-C, it significantly induced G1-arrest in Kelly and SH-SY5Y by 13.6% (*p *= 0.0042) and 17.5% (*p *= 0.0181), respectively (Figure 3A).

To further assess the ability of *miR-323a-3p* to induce apoptosis, we transfected Kelly, SH-SY5Y and BE(2)-C with NC or *miR-323a-3p* mimics and determined the levels of apoptotic markers PARP-cleavage and BCL2 on western blot. The western blot analysis revealed PARP-cleavage in Kelly, SH-SY5Y and BE(2)-C by 197% (*p *= 0.0443), 671% (*p *= 0.0365) and 175% (*p *= 0.0079), respectively, as compared to NC transfected cells (Figure 3B). Moreover, we also observed a reduction in protein levels of BCL2 in Kelly, SH-SY5Y and BE(2)-C by 72% (*p* = 0.0017), 44% (*p* = 0.0007) and 76% (*p* = 0.0024), respectively, as compared to NC transfected cells (Figure 3C). BCL2 mRNA levels were also reduced (Supplementary Figure S3). Taken together, we show that *miR-323a-3p* reduces cell viability by inducing G1-cell cycle arrest and apoptosis in neuroblastoma cells.

### miR-323a-3p targets STAT3 in neuroblastoma

We used bioinformatics target prediction algorithm miRDB to find mRNA binding sequences for *miR-323a-3p.* The miRDB database revealed 793 predicted targets for *miR-323a-3p*. Additionally, a literature search was performed to check previously validated targets of *miR-323a-3p* in other cancers (Supplementary Figure S4A). We used RT-qPCR to scan through a subset of these mRNAs in Kelly cells transfected with *miR-323a-3p* mimics. Compared to NC mimic transfected cells, we observed several mRNAs that were downregulated. *STAT3*, which has not previously been validated as a direct target of *miR-323a-3p*, was consistently downregulated by more than 40% (Supplementary Figure S4B).

**Figure 4:**
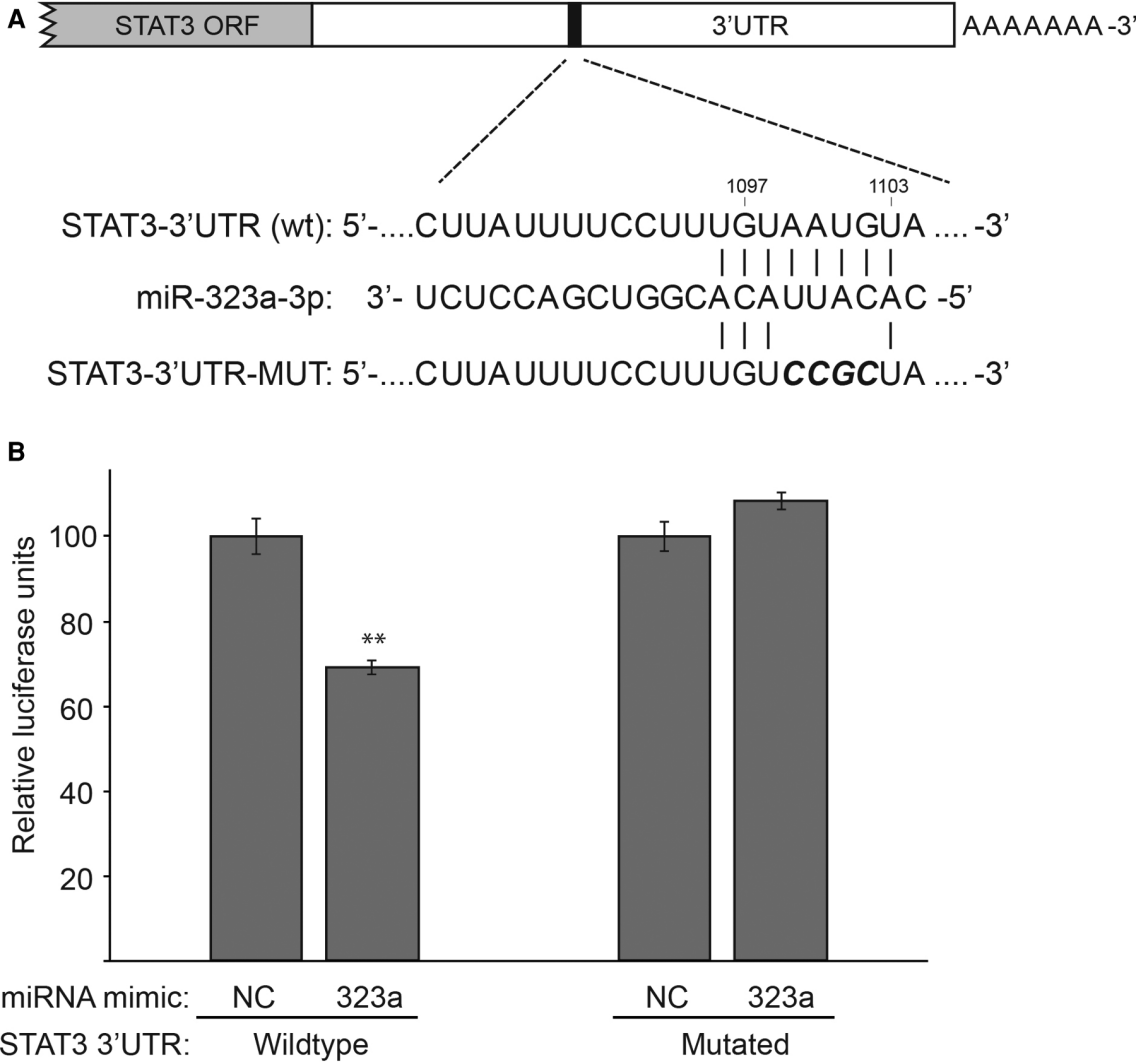
*STAT3* is a direct target of *miR-323a-3p* in neuroblastoma. (**A)** The putative binding site of *miR-323a-3p* (nucleotides 1,097 to 1,103) in the 3′UTR of *STAT3* was mutated as shown in the figure with bold and italics. **(B)** Dual-luciferase reporter assay demonstrating the luciferase activity of a construct with a wild-type or a mutated 3′UTR of *STAT*3 in SH-SY5Y transfected with *miR-323a-3p* or NC mimics. Data is presented as mean ± SD of three independent experiments, each repeated in triplicates. **P < 0.01 vs. the NC. SD, standard deviation; miR, microRNA; NC, negative control; STAT3, signal transducer and activator of transcription; ORF, open Reading frame; UTR, untranslated region; wt, wild-type; MUT, mutated.

miRDB database identified a putative binding site for *miR-323a-3p* in the 3′UTR of *STAT3* (Figure 4A). Thus, to confirm that *STAT3* is a direct target of *miR-323a-3p*, we performed luciferase reporter assay by co-transfecting luciferase construct containing wild-type or mutant 3′UTR of *STAT3* with *miR-323a-3p* or NC mimics. The results showed that overexpression of *miR-323a-3p* suppressed the luciferase activity of wild-type construct by 30.7% (*p *= 0.0014), but not the mutant construct, in SH-SY5Y cells (Figure 4B). Together, these data demonstrated that *STAT3* is a direct target of *miR-323a-3p* in neuroblastoma.

### STAT3 mRNA and protein levels are regulated by miR-323a-3p

**Figure 5:**
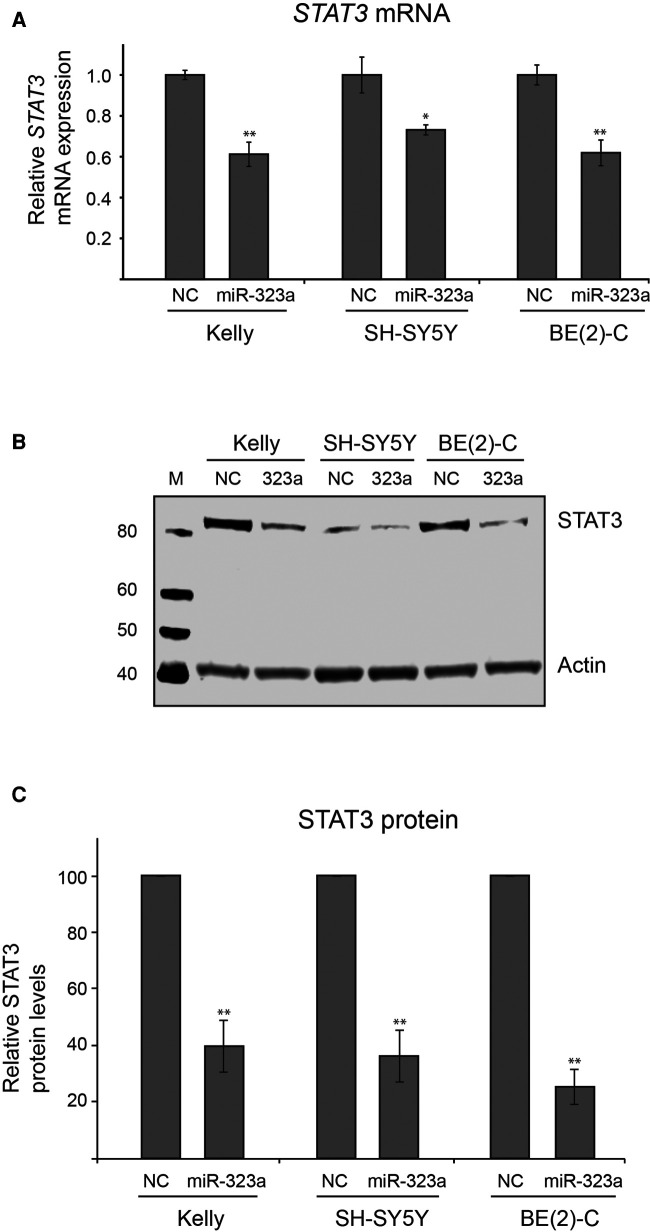
*miR-323a-3p* reduces mRNA and protein levels of STAT3 in neuroblastoma cells. **(A)** The RT-qPCR analysis of *STAT3* mRNA levels in Kelly, SH-SY5Y and BE(2)-C cell lines transfected with *miR-323a-3p.* Data is presented as mean ± SEM of three independent experiments, each repeated in triplicates. **(B)** Western blot assay demonstrating STAT3 protein levels in Kelly, SH-SY5Y and BE(2)-C cell lines transfected with *miR-323a-3p*. **(C)** Quantification of STAT3 protein expression on the western blots (*n* = 3). Data is presented as mean ± SD of three independent experiments. *P < 0.05, **P < 0.01 vs. the NC. RT-qPCR, reverse transcription-quantitative polymerase chain reaction; SD, standard deviation; miR, microRNA; NC, negative control; STAT3, signal transducer and activator of transcription.

We next investigated whether *miR-323a-3p* could regulate *STAT3* at mRNA and protein levels. The *miR-323a-3p* or NC mimics were transfected into neuroblastoma cell lines and the expression levels of *STAT3* mRNA and protein were examined by RT-qPCR and western blot analysis, respectively. Overexpression of *miR-323a-3p* led to significant decrease of *STAT3* mRNA in Kelly, SH-SY5Y and BE(2)-C by 42% (*p *= 0.0049), 31% (*p *= 0.0137) and 43% (*p *= 0.0039), respectively, as compared to NC transfected cells (Figure 5A). Moreover, STAT3 protein levels were also significantly decreased upon *miR-323a-3p* overexpression in Kelly, SH-SY5Y and BE(2)-C by 60% (*p *= 0.0079), 64% (*p *= 0.0070) and 75% (*p *= 0.0023), respectively, as compared to NC transfected cells (Figures 5B, C). Altogether, these data suggest that *miR-323a-3p* directly binds and inhibits the expression of *STAT3* mRNA and protein levels in neuroblastoma cells.

## Discussion

Analyzing neuroblastoma tumor data of *miR-323a-3p* revealed that this miRNA is reverse correlated with MNA and high-stage disease and that there is a significant association between low *miR-323a* levels and poor overall survival.

The *miR-323a-3p* plays a significant role in tumorigenesis by regulating various genes, and the mechanism of action of this miRNA differs from cancer to cancer (13–19). Observing low expression of *miR-323a* in neuroblastoma cell lines from patients with relapsed neuroblastoma and tumors from high-risk patients led us to hypothesize that overexpression of *miR-323a-3p* could have positive phenotypic effect on neuroblastoma cell lines. Indeed, cell viability was clearly reduced upon transient transfection with *miR-323a-3p* due to G1-arrest and apoptosis. Compared to the Kelly and SH-SY5Y cell lines, transfection with *miR-323a-3p* caused less reduction of cell viability in BE(2)-C, and failed to induce significant G1-cell cycle arrest in this cell line. This might be explained by lower transfection efficiency in the BE(2)-C cell line (Supplementary Figure S2), in addition to a higher pre-existing expression level (Figure 2A). Nonetheless, our study suggests a tumor suppressive role of *miR-323a-3p* in neuroblastoma, which coincides with most of the studies conducted in other cancer types (13–15, 18). It is worth noting that, in contrast to these studies, *miR-323* was upregulated and shown to promote cell proliferation and growth of xenograft tumors by targeting *p73* in prostate cancer (19, 36). However, the authors do not provide information on which miRNA (miR-323a or -b) or which mature miRNA (-3p or -5p) they have investigated. If they indeed investigated *miR-323a-3p,* the conflicting results merely demonstrate the dual roles of miRNAs. MiRNA's ability to regulate several targets within a cell can produce different phenotypes in different cells and diseases, and even within the same disease (7, 37). Therefore, understanding the cell or disease specific mechanisms of a miRNA through functional studies is imperative to develop targeted therapies.

Further elucidating the role of *miR-323a-3p*, we searched for its unknown targets. Expression of a selection of genes involved in proliferation, cell cycle and apoptosis were screened using RT-qPCR in neuroblastoma cells transfected with either *miR-323a-3p* or negative control mimics. *STAT3*, a gene well known to function in apoptosis (22) and that induces G1-arrest when silenced in esophageal carcinoma (23), was significantly reduced by *miR-323a-3p* in all three cell lines, and a functional binding site for *miR-323a-3p* in the STAT3 3´UTR was confirmed by luciferase assay. Although other genes that could contribute to the observed biological effects mediated by exogenous expression of *miR-323a-3p* were observed downregulated (particularly *SMAD2*, *TGFA* and *TGFB2*), the levels of these were low (Cq-values >31) and therefore not studied further.

When analyzing non-MNA neuroblastoma tumor data, we did not observe a correlation between STAT3 and *miR-323a-3p* expression (data not shown). This underscores the importance of conducting further investigations to establish if the phenotypic effect we observe from upregulation of *miR-323a-3p* is indeed functionally linked to inhibition of *STAT3*. There are undoubtably other mRNA targets for *miR-323a-3p* that can cause the loss of viability and apoptosis that we observe. Crosslink immunoprecipitation followed by high-throughput sequencing to detect miRNA-mRNA interactions could potentially have generated more targets to investigate.

*MYCN* amplification is one of the most powerful biological markers indicating poor prognosis in neuroblastoma (38). Analyzing tumor data, we see a lower *miR-323a-3p* expression in MNA tumors compared to non-MNA tumors, further supporting the assumption that *miR-323a-3p* is a tumor suppressor in this malignancy. Basic expression of *miR-323a-3p* in the three cell lines we have tested do not coincide with the tumor data. Considering the heterogeneous nature of neuroblastoma, variations between single cell lines will occur. Various biological factors, like chromosomal aberrations or gene mutations can alter the miRNA expression, thus accounting for the higher expression in BE(2)-C. As the number of cell lines in this study is limited, generalization of gene expression is restricted. Furthermore, compared to its isogenic counterpart, SK-N-BE(1), expression of *miR-323a-3p* in BE(2)-C is indeed downregulated (7).

The documented role of miRNAs to act as either oncogenes (oncomiRs) or tumor suppressor genes in multiple cancers has led to clinical trials aiming to reconstitute downregulated tumor suppressor miRNAs or inhibit highly expressed miRNAs. *MiR-34* (MRX34) has been tested in a phase-I clinical trial (ClinicalTrials.gov Identifier: NCT01829971) for treating solid tumors and *miR-122* entered phase-II trial (ClinicalTrials.gov Identifier: NCT01200420) for treating hepatitis (39–41). Thus, strategies involving manipulating expression of miRNAs can be an important approach in treatment of cancers or other diseases. As our study suggests a tumor suppressive function of *miR-323a-3p* in neuroblastoma, it is intriguing to consider it valid for further testing. Although the number of cell lines used limits our study, it is indicative of the biological functions of *miR-323a-3p* in neuroblastoma and provides novel knowledge about the neuroblastoma targetome.

## Conclusions

In conclusion, our study provides new insights into the functional roles of *miR-323a-3p* in neuroblastoma. We demonstrate that *miR-323a-3p* is downregulated in tumors with high-risk features. Moreover, ectopic expression of *miR-323a-3p* in neuroblastoma cell lines lead to reduced cell viability, G1-cell cycle arrest and apoptosis, and caused reduced expression of *STAT3* because of direct binding of *miR-323a-3p* to the 3′UTR of *STAT3* mRNA.

## Data Availability

The raw data supporting the conclusions of this article will be made available by the authors, without undue reservation.

## References

[1] Wagner LM Danks MK. New therapeutic targets for the treatment of high-risk neuroblastoma. J Cell Biochem. (2009) 107(1):46–57. 10.1002/jcb.22094. 19277986

[2] Smith V Foster J. High-Risk neuroblastoma treatment review. Children (Basel). (2018) 5(9). 10.3390/children5090114 PMC6162495 30154341

[3] Pugh TJ Morozova O Attiyeh EF Asgharzadeh S Wei JS Auclair D The genetic landscape of high-risk neuroblastoma. Nat Genet. (2013) 45(3):279–84. 10.1038/ng.2529. 23334666 PMC3682833

[4] O'Brien J Hayder H Zayed Y Peng C. Overview of MicroRNA biogenesis. Mechanisms of Actions, and Circulation. Front Endocrinol (Lausanne). (2018) 9:402. 10.3389/fendo.2018.00402. 30123182 PMC6085463

[5] Pavlikova L Seres M Breier A Sulova Z. The roles of microRNAs in cancer multidrug resistance. Cancers (Basel). (2022) 14(4). 35205839 10.3390/cancers14041090 PMC8870231

[6] Ayers D Mestdagh P Van Maerken T Vandesompele J. Identification of miRNAs contributing to neuroblastoma chemoresistance. Comput Struct Biotechnol J. (2015) 13:307–19. 10.1016/j.csbj.2015.04.003. 25973145 PMC4427660

[7] Roth SA Knutsen E Fiskaa T Utnes P Bhavsar S Hald OH Next generation sequencing of microRNAs from isogenic neuroblastoma cell lines isolated before and after treatment. Cancer Lett. (2016) 372(1):128–36. 10.1016/j.canlet.2015.11.026. 26708804

[8] Baker DL Schmidt ML Cohn SL Maris JM London WB Buxton A Outcome after reduced chemotherapy for intermediate-risk neuroblastoma. N Engl J Med. (2010) 363(14):1313–23. 10.1056/NEJMoa1001527. 20879880 PMC2993160

[9] Gonzalez-Vallinas M Rodriguez-Paredes M Albrecht M Sticht C Stichel D Gutekunst J Epigenetically regulated chromosome 14q32 miRNA cluster induces metastasis and predicts poor prognosis in lung adenocarcinoma patients. Mol Cancer Res. (2018) 16(3):390–402. 10.1158/1541-7786.MCR-17-0334. 29330288

[10] Nadal E Zhong J Lin J Reddy RM Ramnath N Orringer MB A MicroRNA cluster at 14q32 drives aggressive lung adenocarcinoma. Clin Cancer Res. (2014) 20(12):3107–17. 10.1158/1078-0432.CCR-13-3348. 24833665

[11] Zehavi L Avraham R Barzilai A Bar-Ilan D Navon R Sidi Y Silencing of a large microRNA cluster on human chromosome 14q32 in melanoma: biological effects of mir-376a and mir-376c on insulin growth factor 1 receptor. Mol Cancer. (2012) 11:44. 10.1186/1476-4598-11-44. 22747855 PMC3444916

[12] Hoshi M Otagiri N Shiwaku HO Asakawa S Shimizu N Kaneko Y Detailed deletion mapping of chromosome band 14q32 in human neuroblastoma defines a 1.1-mb region of common allelic loss. Br J Cancer. (2000) 82(11):1801–7. 10.1054/bjoc.2000.1108. 10839294 PMC2363232

[13] Shahar T Granit A Zrihan D Canello T Charbit H Einstein O Expression level of miRNAs on chromosome 14q32.31 region correlates with tumor aggressiveness and survival of glioblastoma patients. J Neurooncol. (2016) 130(3):413–22. 10.1007/s11060-016-2248-0. 27573219

[14] Chen H Gao S Cheng C. MiR-323a-3p suppressed the glycolysis of osteosarcoma via targeting LDHA. Hum Cell. (2018) 31(4):300–9. 10.1007/s13577-018-0215-0. 30088225

[15] Wang C Liu P Wu H Cui P Li Y Liu Y MicroRNA-323-3p inhibits cell invasion and metastasis in pancreatic ductal adenocarcinoma via direct suppression of SMAD2 and SMAD3. Oncotarget. (2016) 7(12):14912–24. 10.18632/oncotarget.7482. 26908446 PMC4924761

[16] Shi P Zhang J Li X Li W Li H Fu P. Long non-coding RNA NORAD inhibition upregulates microRNA-323a-3p to suppress tumorigenesis and development of breast cancer through the PUM1/eIF2 axis. Cell Cycle. (2021) 20(13):1295–307. 10.1080/15384101.2021.1934627. 34125645 PMC8331030

[17] Xu XH Song W Li JH Huang ZQ Liu YF Bao Q Long non-coding RNA EBLN3P regulates UHMK1 expression by sponging miR-323a-3p and promotes colorectal cancer progression. Front Med (Lausanne). (2021) 8:651600. 10.3389/fmed.2021.651600. 34109193 PMC8180563

[18] Li J Xu X Meng S Liang Z Wang X Xu M MET/SMAD3/SNAIL circuit mediated by miR-323a-3p is involved in regulating epithelial-mesenchymal transition progression in bladder cancer. Cell Death Dis. (2017) 8(8):e3010. 10.1038/cddis.2017.331. 28837140 PMC5596538

[19] Gao Q Yao X Zheng J. MiR-323 inhibits prostate cancer vascularization through adiponectin receptor. Cell Physiol Biochem. (2015) 36(4):1491–8. 10.1159/000430313. 26160610

[20] Copeland NG Gilbert DJ Schindler C Zhong Z Wen Z Darnell JE Jr. Distribution of the mammalian stat gene family in mouse chromosomes. Genomics. (1995) 29(1):225–8. 10.1006/geno.1995.1235 8530075

[21] Darnell JE Jr. STATs and gene regulation. Science. (1997) 277(5332):1630–5. 10.1126/science.277.5332.1630 9287210

[22] Yu H Lee H Herrmann A Buettner R Jove R. Revisiting STAT3 signalling in cancer: new and unexpected biological functions. Nat Rev Cancer. (2014) 14(11):736–46. 10.1038/nrc3818. 25342631

[23] Zhou C Ma J Su M Shao D Zhao J Zhao T Down-regulation of STAT3 induces the apoptosis and G1 cell cycle arrest in esophageal carcinoma ECA109 cells. Cancer Cell Int. (2018) 18:53. 10.1186/s12935-018-0549-4. 29636641 PMC5883295

[24] Sugimura K Miyata H Tanaka K Hamano R Takahashi T Kurokawa Y Let-7 expression is a significant determinant of response to chemotherapy through the regulation of IL-6/STAT3 pathway in esophageal squamous cell carcinoma. Clin Cancer Res. (2012) 18(18):5144–53. 10.1158/1078-0432.CCR-12-0701. 22847808

[25] Zhuang G Wu X Jiang Z Kasman I Yao J Guan Y Tumour-secreted miR-9 promotes endothelial cell migration and angiogenesis by activating the JAK-STAT pathway. EMBO J. (2012) 31(17):3513–23. 10.1038/emboj.2012.183. 22773185 PMC3433782

[26] Du L Subauste MC DeSevo C Zhao Z Baker M Borkowski R miR-337-3p and its targets STAT3 and RAP1A modulate taxane sensitivity in non-small cell lung cancers. PLoS One. (2012) 7(6):e39167. 10.1371/journal.pone.0039167. 22723956 PMC3377607

[27] Yang X Liang L Zhang XF Jia HL Qin Y Zhu XC MicroRNA-26a suppresses tumor growth and metastasis of human hepatocellular carcinoma by targeting interleukin-6-Stat3 pathway. Hepatology. (2013) 58(1):158–70. 10.1002/hep.26305. 23389848

[28] Navarro A Diaz T Martinez A Gaya A Pons A Gel B Regulation of JAK2 by miR-135a: prognostic impact in classic hodgkin lymphoma. Blood. (2009) 114(14):2945–51. 10.1182/blood-2009-02-204842. 19666866

[29] Liu W Wang X. Prediction of functional microRNA targets by integrative modeling of microRNA binding and target expression data. Genome Biol. (2019) 20(1):18. 10.1186/s13059-019-1629-z. 30670076 PMC6341724

[30] Bhavsar SP Lokke C Flaegstad T Einvik C. Hsa-miR-376c-3p targets cyclin D1 and induces G1-cell cycle arrest in neuroblastoma cells. Oncol Lett. (2018) 16(5):6786–94. 30405823 10.3892/ol.2018.9431 PMC6202480

[31] Untergasser A Ruijter J Benes V van den Hoff MJB. Web-based LinRegPCR: application for the visualization and analysis of (RT)-qPCR amplification and melting data. BMC Bioinform. (2021) 22(1):398. 10.1186/s12859-021-04306-1. PMC8386043 34433408

[32] Ramakers C Ruijter JM Deprez RH Moorman AF. Assumption-free analysis of quantitative real-time polymerase chain reaction (PCR) data. Neurosci Lett. (2003) 339(1):62–6. 10.1016/S0304-3940(02)01423-4. 12618301

[33] Taylor SC Nadeau K Abbasi M Lachance C Nguyen M Fenrich J. The ultimate qPCR experiment: producing publication quality, reproducible data the first time. Trends Biotechnol. (2019) 37(7):761–74. 10.1016/j.tibtech.2018.12.002. 30654913

[34] Schindelin J Arganda-Carreras I Frise E Kaynig V Longair M Pietzsch T Fiji: an open-source platform for biological-image analysis. Nat Methods. (2012) 9(7):676–82. 10.1038/nmeth.2019. 22743772 PMC3855844

[35] Therneau TM Grambsch PM. Modeling survival data: Extending the cox model. New York: Springer (2000. ISBN 0-387-98784-3.

[36] Gao Q Zheng J. microRNA-323 upregulation promotes prostate cancer growth and docetaxel resistance by repressing p73. Biomed Pharmacother. (2018) 97:528–34. 10.1016/j.biopha.2017.10.040. 29091904

[37] Roth SA Hald OH Fuchs S Lokke C Mikkola I Flaegstad T MicroRNA-193b-3p represses neuroblastoma cell growth via downregulation of cyclin D1, MCL-1 and MYCN. Oncotarget. (2018) 9(26):18160–79. 10.18632/oncotarget.24793. 29719597 PMC5915064

[38] Matthay KK Maris JM Schleiermacher G Nakagawara A Mackall CL Diller L Neuroblastoma. Nat Rev Dis Primers. (2016) 2(16078). 27830764 10.1038/nrdp.2016.78

[39] Bouchie A. First microRNA mimic enters clinic. Nat Biotechnol. (2013) 31(7):577. 10.1038/nbt0713-577. 23839128

[40] Janssen HL Reesink HW Lawitz EJ Zeuzem S Rodriguez-Torres M Patel K Treatment of HCV infection by targeting microRNA. N Engl J Med. (2013) 368(18):1685–94. 10.1056/NEJMoa1209026. 23534542

[41] Beg MS Brenner AJ Sachdev J Borad M Kang YK Stoudemire J Phase I study of MRX34, a liposomal miR-34a mimic, administered twice weekly in patients with advanced solid tumors. Invest New Drugs. (2017) 35(2):180–8. 10.1007/s10637-016-0407-y. 27917453 PMC5893501

