# Supplementary material for: Hsa-miR-323a-3p functions as a tumor suppressor and targets STAT3 in neuroblastoma cells
Source: Front Pediatr. 2023 Mar 24;11:1098999. doi: 10.3389/fped.2023.1098999 (PMC10079869; doi:10.3389/fped.2023.1098999)
Supplement: Supplementary file 1 [file Datasheet1.pdf]

Supplementary Material

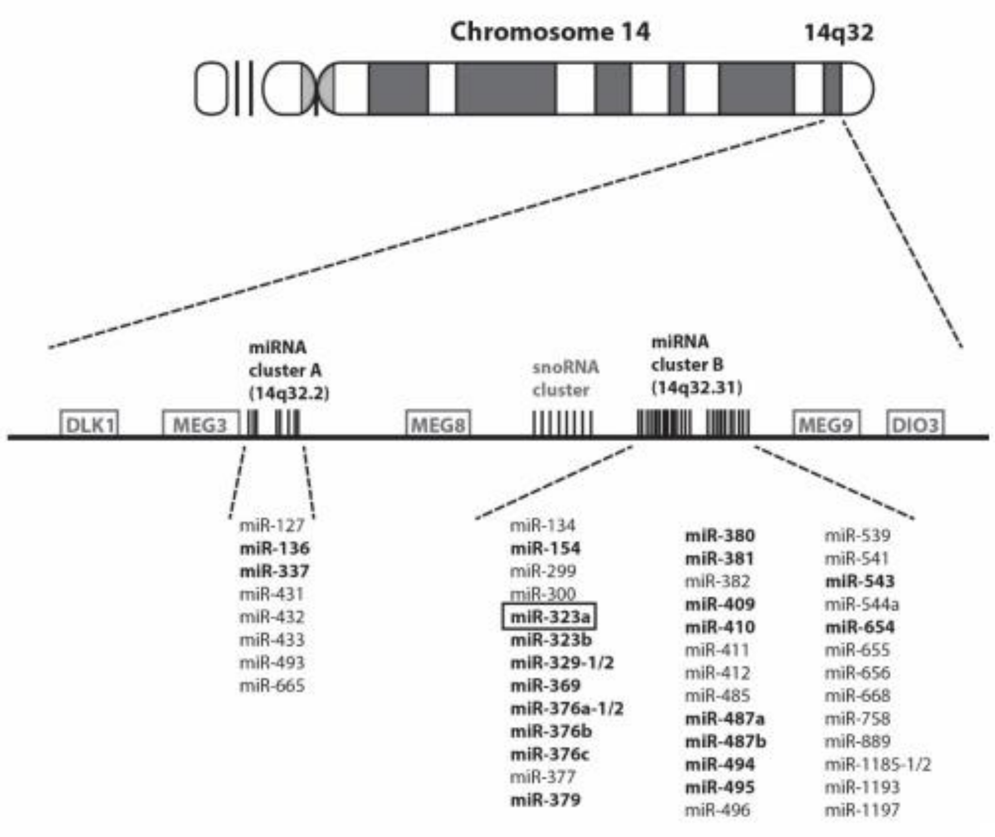

Supplementary figure S1: *MIR-323a,* located on chromosome 14q32, is differentially expressed in neuroblastoma cell line pairs. The miRNAs located on chromosome 14q32 region are upregulated or downregulated (bold type) in neuroblastoma cell line pairs. The miRNA of interest, *miR-323a,* is highlighted in rectangular box. MEG, Maternally expressed; SnoRNA, Small nucleolar RNAs; DLK1, Delta like non-canonical notch ligand 1; DIO3, Iodothyronine deiodinase 3; miR, microRNA.

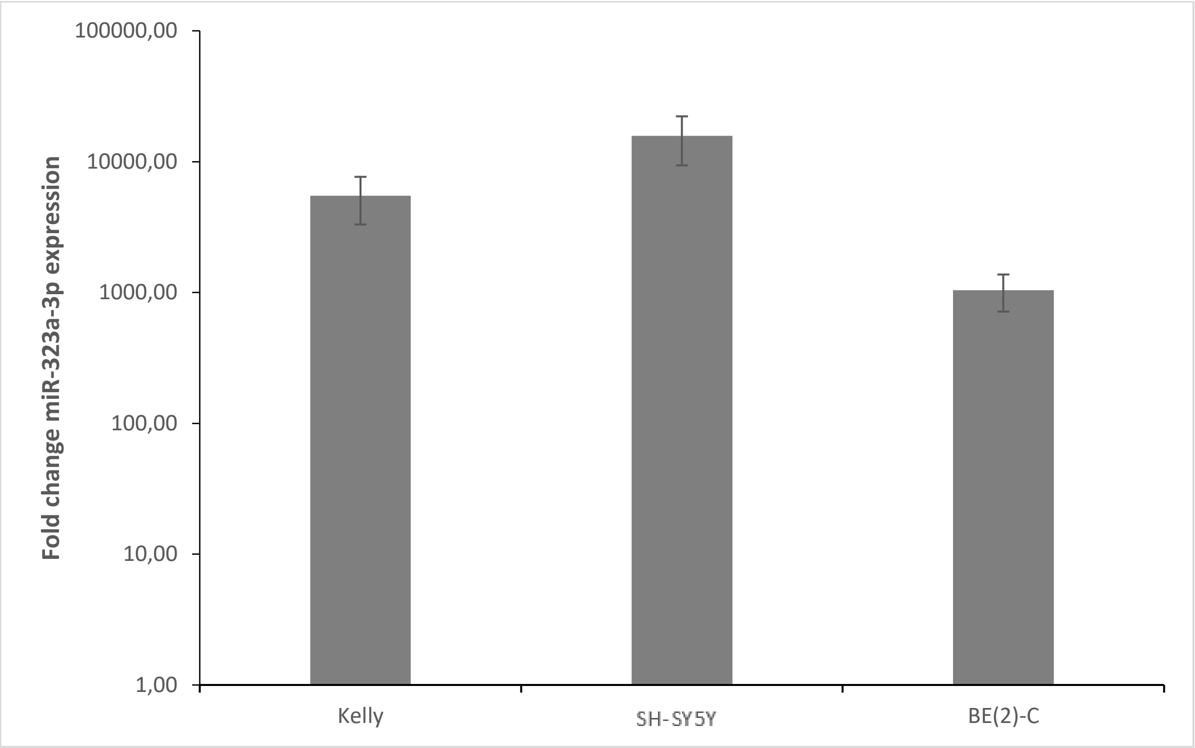

Supplementary figure S2: Transfection efficiency of *miR-323a-3p* in neuroblastoma cells. RT-qPCR analysis for confirmation of *miR-323a-3p* overexpression in Kelly, SH-SY5Y and BE(2)-C cell lines transfected with NC or *miR-323a-3p*. The expression of *miR-323a-3p* in NC transfected cells was set to 1 and *miR-4286* served as an endogenous control for miRNAs. Data are presented in log scale as mean ± SD of two independent experiments, each repeated in triplicates.

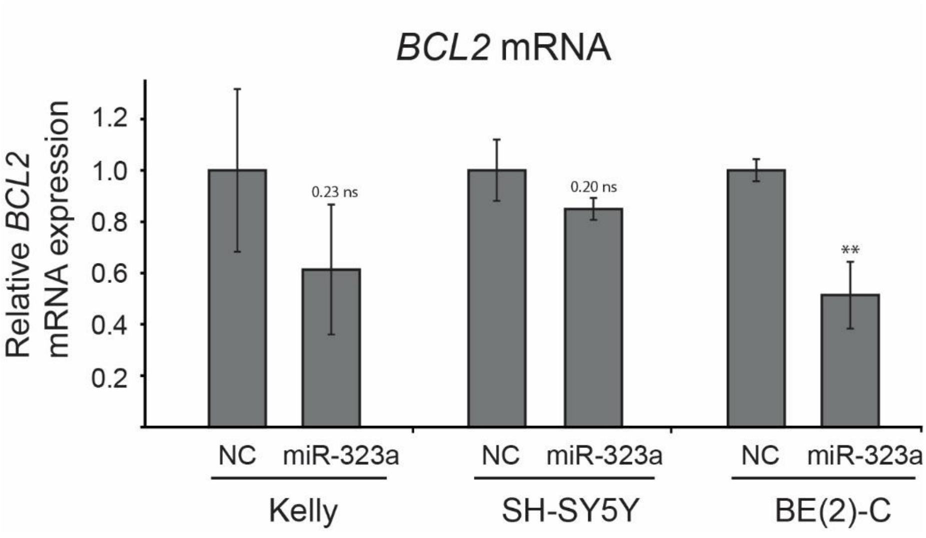

Supplementary figure S3: The RT-qPCR analysis of *BCL2* mRNA levels in Kelly, SH-SY5Y and BE(2)-C cell lines transfected with *miR-323a-3p*. Data is presented as mean ± SEM of three independent experiments, each repeated in triplicates. **P<0.01 vs. the NC. RT-qPCR, reverse transcription-quantitative polymerase chain reaction; ns, non-significant; SD, standard deviation; miR, microRNA; NC, negative control; BCL2, B-cell lymphoma 2.

A

miRNA	Gene	Gene description	Pred.	Valid.	Refs.
hsa-miR-323a-3p	BMP1	Bone morphogenetic protein 1	X		miRDB
hsa-miR-323a-3p	BMP3	Bone morphogenetic protein 3	X		miRDB
hsa-miR-323a-3p	BRI3	Brain protein I3		X	(1, 2)
hsa-miR-323a-3p	CDK6	Cyclin dependent kinase 6		X	(2)
hsa-miR-323a-3p	CDKN1B	Cyclin dependent kinase inhibitor 1B	X		miRDB
hsa-miR-323a-3p	CDK19	Cyclin dependent kinase 19	X		miRDB
hsa-miR-323a-3p	SMAD2	SMAD family member 2		X	(3-5)
hsa-miR-323a-3p	SMAD3	SMAD family member 3		X	(4, 5)
hsa-miR-323a-3p	SMAD5	SMAD family member 5	X		miRDB
hsa-miR-323a-3p	TGFA	Transforming growth factor alpha		X	(3)
hsa-miR-323a-3p	TGFB2	Transforming growth factor beta 2	X		miRDB
hsa-miR-323a-3p	STAT3	Signal transducer and activator of transcription 3	X		miRDB

Abbreviations: Pred., predicted; Valid., validated; Refs., references

B

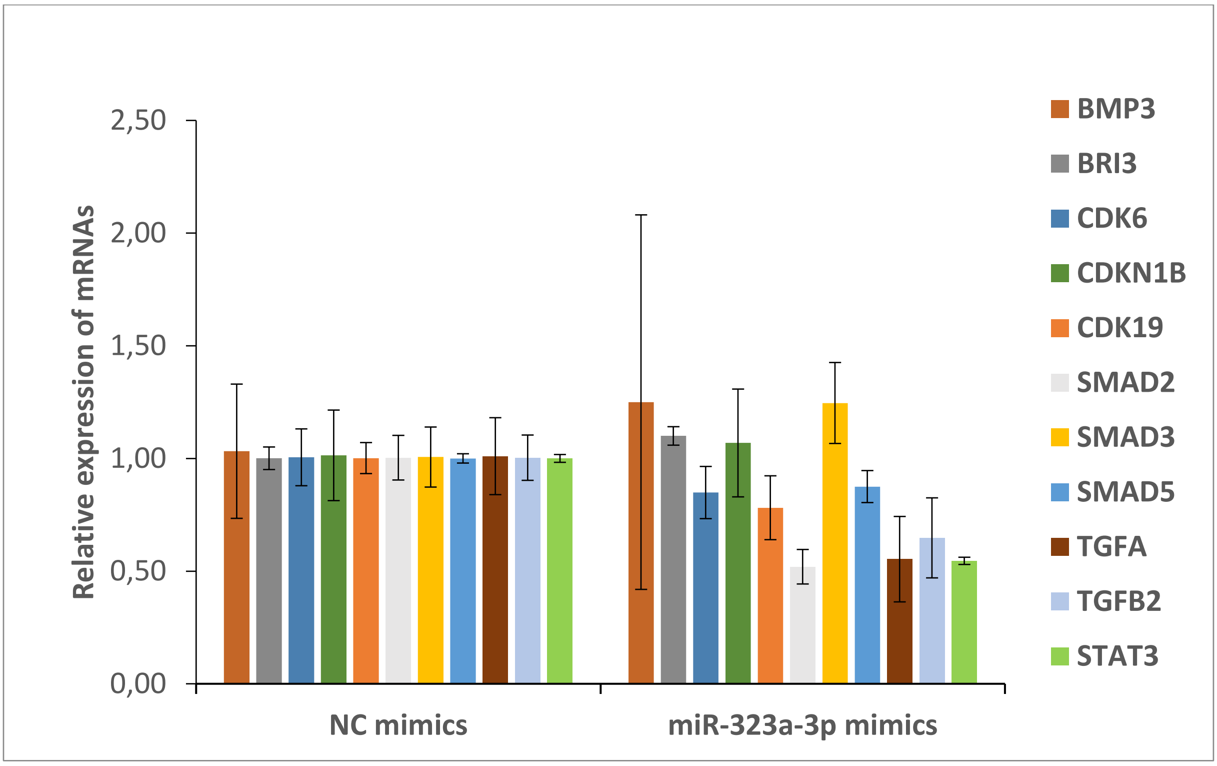

Supplementary figure S4: Screening of selected *miR-323a-3p* targets in the cell line Kelly. **A)** Selected list of predicted or validated (as direct targets by luciferase 3'UTR assay) targets of *miR-323a-3p*. **B)** RT-qPCR analysis for screening of *miR-323a-3p* targets in Kelly cell line transfected with NC or *miR-323a-3p* mimics. The expression of *miR-323a-3p* in NC transfected cells was set to 1 and *miR-4286* served as an endogenous control for miRNAs. Data are presented as mean ± SD of each experiment repeated in triplicates.

References for Supplementary Figure S4

1. Yang L, Xiong Y, Hu X-F, Du Y-H. MicroRNA-323 regulates ischemia/reperfusion injury-induced neuronal cell death by targeting BRI3. International journal of clinical and experimental pathology. 2015;8(9):10725-33.

2. Zhang H, Wang X, Chen X. Potential Role of Long Non-Coding RNA ANRIL in Pediatric Medulloblastoma Through Promotion on Proliferation and Migration by Targeting miR-323. Journal of Cellular Biochemistry. 2017;118(12):4735-44.

3. Ge L, Habiel DM, Hansbro PM, Kim RY, Gharib SA, Edelman JD, et al. miR-323a-3p regulates lung fibrosis by targeting multiple profibrotic pathways. JCI insight. 2016;1(20):e90301-e.

4. Kärner J, Wawrzyniak M, Tankov S, Runnel T, Aints A, Kisand K, et al. Increased microRNA-323-3p in IL-22/IL-17-producing T cells and asthma: a role in the regulation of the TGF-β pathway and IL-22 production. Allergy. 2017;72(1):55-65.

5. Wang C, Liu P, Wu H, Cui P, Li Y, Liu Y, et al. MicroRNA-323-3p inhibits cell invasion and metastasis in pancreatic ductal adenocarcinoma via direct suppression of SMAD2 and SMAD3. Oncotarget. 2016;7(12):14912-24.
